# Alcohol Abuse and Insomnia Disorder: Focus on a Group of Night and Day Workers

**DOI:** 10.3390/ijerph182413196

**Published:** 2021-12-14

**Authors:** Fulvio Plescia, Luigi Cirrincione, Daniela Martorana, Caterina Ledda, Venerando Rapisarda, Valentina Castelli, Francesco Martines, Denis Vinnikov, Emanuele Cannizzaro

**Affiliations:** 1 Department of Health Promotion Sciences Maternal and Child Care, Internal Medicine and Medical Specialties “Giuseppe D’Alessandro”, University of Palermo, Via del Vespro 133, 90127 Palermo, Italy; luigicirrincione@gmail.com; 2 Department of Orthopaedic Surgery, Azienda Ospedaliera “Ospedali Riuniti Villa Sofia Cervello”, 90146 Palermo, Italy; dani.m@hotmail.it; 3 Department of Clinical and Experimental Medicine, Occupational Medicine, University of Catania, 95123 Catania, Italy; cledda@unict.it (C.L.); vrapisarda@unict.it (V.R.); 4 Department of Biomedicine, Neuroscience and Advanced Diagnostics (BiND), Section of Audiology, University of Palermo, Via del Vespro, 129, 90127 Palermo, Italy; valentina.castelli02@unipa.it (V.C.); francesco.martines@unipa.it (F.M.); 5 Faculty of Medicine and Healthcare, Al-Farabi Kazakh National University, Al-Farabi Avenue 71, Almaty 050040, Kazakhstan; denisvinnikov@mail.ru

**Keywords:** alcohol, sleep disorders, night workers, AUDIT-C

## Abstract

The sleep-wake cycle plays a fundamental role in maintaining the physiological balance of our body. Its alteration favours the genesis of several organic alterations and diseases including sleep disorders and the consumption of several substances of abuse. It has been reported that the work activity, especially that carried out during the night, is able to influence the sleep-wake cycle, promoting the development of insomnia, which, in turn, would subject the worker to a stressful condition such as to encourage adverse behaviour such as the use/abuse of psychotropic substances. Based on the above premises, the aim of our research was to evaluate, in night workers: (i) the pattern of consumption of alcoholic beverages; (ii) the presence of insomnia; and (iii) the possible correlation between alcohol consumption and insomnia disorder. We used the AUDIT-C test (the abbreviated version of the Alcohol Use Disorders Identification Test) and the Insomnia Severity Index to assess alcohol consumption and insomnia disorder, respectively. All questionnaires were completed by workers of both sexes belonging to different types of work activities, exclusively day or night. The results of our research show a higher propensity of night workers to consume alcoholic beverages than those who work during daytime hours, often in binge-drinking mode. In addition, an increase in the amount of alcohol consumed was found to be related to insomnia disorder, especially in night workers. This study provides further awareness of the importance of the negative impact of alcohol consumption on sleep quality in night workers.

## 1. Introduction

The sleep-wake cycle plays a key role in the genesis of several mental disorders such as post-traumatic stress disorder (PTDS), straining, depression, anxiety, and is also associated with sleep disorders and substance abuse. [1,2,3,4,5,6]. Sleep disorders include disturbances in sleep initiation and sleep maintenance which, in turn, may adversely affect chronic disease outcomes by increasing related mortality and morbidity [7,8,9,10,11,12]. For example, obstructive sleep apnea syndrome, now recognized as one of the most frequent causes of excessive daytime sleepiness, has been identified as a contributing or contributing factor or cofactor in a significant number of work-related accidents [13].

It has been reported that there is a bidirectional relationship between sleep disorders and serious medical conditions, which are commonly related to work and age. People suffering from sleep disorders are usually more prone to develop hypertension, depression, cardiovascular and cerebrovascular diseases; conversely, individuals with any of these clinical conditions or with olfactory dysfunction and chronic nasal disorders are at a higher risk than healthy individuals of developing sleep-related problems [14,15,16,17,18,19]. In this regard, different types of work activities seem to influence the onset of diseases related to an alteration of the sleep-wake cycle, also in relation to the continuous increase in stress conditions, of the work environment, related to the recent pandemic from Sars-CoV 2 that has also invested the world of work [20,21,22,23].

In particular, night work (all work activities performed during a period of no less than three consecutive hours, from midnight to 5:00 a.m.) seems to have harmful effects on people’s ability to adapt to the natural cycles of light and darkness and, consequently, could play a critical role in the genesis of several health problems such as cardiovascular diseases, gastrointestinal diseases and sleep disorders [24,25,26]. Regarding this, it is worth mentioning that night work and work-related stress are assessed in the Risk Assessment Document with a combined multidisciplinary approach and are the subject of health promotion campaigns and periodic training in the workplace [27,28,29].

In recent years, many researchers have focused on the relationship between sleep disorders and drug abuse. Research in adults indicates that sleep disorders, particularly insomnia, are related to stress and are commonly associated with substance abuse (cigarettes, alcohol, and illicit drugs), which, in turn, can affect sleep architecture by altering night-time awakenings and deeper sleep. Furthermore, the association between substance abuse and sleep disorders appears to be synchronous, with sleep problems increasing the risk of developing substance use disorders, and acute and chronic substance use potentially leading to sleep disorders [30,31,32,33,34,35]. Several studies pay particular attention to differences in risk factors, such as weight gain, dyslipidaemia, and substance abuse, between shift/night workers and day workers, which may contribute to the onset of various diseases [35,36,37,38,39]. Among these risk factors, alcohol consumption seems to be positively related to shift work activity, and it also appears that shift workers are likely to be binge-drinkers [40,41,42]. However, some studies have revealed no difference in alcohol consumption patterns between night/shift workers and daily workers [43,44].

Alcohol, especially through its metabolites including acetaldehyde, is known to be a central nervous system depressant, with multiple effects including sedative effects, memory and learning deficits, and sleep-wake cycle disturbances [45,46]. Low doses of alcohol promote sleep induction, whereas, when its consumption becomes excessive, it reduces sleep quality [47]. In addition, large amounts of alcohol are associated with an increase in sleep latency, total sleep time and affect REM (rapid eye movement) sleep [48,49].

Based on the above premises, the purpose of this study was to firstly assess the association between night work and alcohol consumption and secondly the effect of alcohol on normal sleep, and the response to its use by people with insomnia disorder. Although this research had a limited sample size, it may make a further contribution to the investigation of the negative effects of night work as a possible risk factor for undesirable health effects and substance abuse.

## 2. Materials and Methods

### 2.1. Design

Data were collected from a private practice of Occupational Medicine in Palermo, Italy. This cross-sectional study was conducted from June 2020 to May 2021.

Subjects of both sexes were enrolled, aged between 24 and 67 years, and with a work history of at least 2 years.

Excluded from the study were all those who, at the time of the medical examination, presented sleep disorders, anxiety and depression or other psychiatric disorders treated with drugs; those with a BMI greater than or equal to 32; those with dysmetabolic diseases such as diabetes, hyperinsulinemia, hypercholesterolemia and hypertriglyceridemia under pharmacological treatment; those with current or previous oncological diseases; and those undergoing immunosuppressive or corticosteroid treatment. In addition, subjects who had been symptomatically infected with COVID-19 in the last six months were excluded from the study. These exclusion criteria were considered on the basis of the influence that both drug therapies and particular living conditions might have on sleep quality.

Specifically, the population consisted of 600 (100%) workers belonging to a different type of daily or night work activity (security guard, mail sorter, production worker, parcel sorter) invited to participate in this study by their management. Among them 10% (n = 60) refused to participate, the study initially recruited 540 (90%) workers. All participants were informed about the purpose of the study and signed informed consent before participating. Respondents were asked not to mention their name or the name of their organization in the questionnaire to ensure privacy and anonymity.

At the first visit, all workers were questioned about their medical history, which included a differential diagnosis of sleep disorders, alcohol, or drug abuse, and underwent a physical examination (blood pressure measurements and electrocardiogram acquired by instrumental assessment from all patients).

At the end of the medical evaluation, a further 5% (30) were excluded from the study because they were undergoing anxiolytic or antidepressant therapy at the time of the research. Finally, subjects with a body mass index greater than 32 with obvious dysmetabolic diseases or undergoing major therapies with antineoplastic drugs, immunosuppressants or corticosteroids or people who had contracted COVID symptomatically, requiring hospitalization were not included 12% (72).

**Figure 1:**
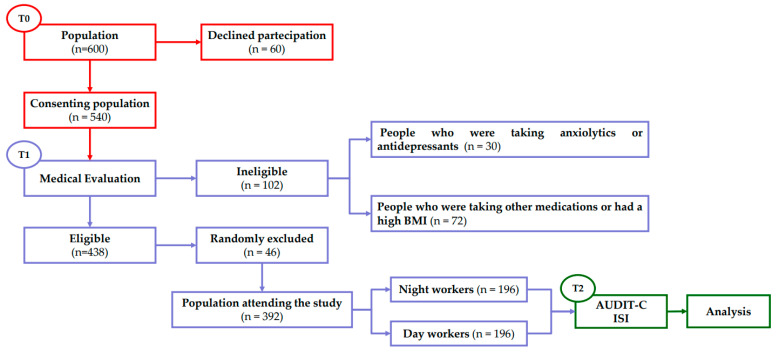
Flow chart of experimental procedures. T0: period in which the population was recruited; T1: period of general patient history useful for determining the final number of the population used in the study; T2: period in which the tests were administered and analysed.

We also randomly excluded an additional 18 males and 28 females to obtain the same number of day or night workers for each male and female sample. In the end, the study enrolled 392 adults, 222 males and 170 females (M/F ratio 1.3) (Table 1), divided into two different groups: 196 day workers (DW) and 196 night workers (NW). At the end of the patients’ general anamnesis, all participants were invited to complete questionnaires regarding alcohol use, and insomnia disturbance (Figure 1).

All data were managed according to the Italian law for the protection of privacy (Decree n. 196, January 2003). A multidisciplinary team of health experts collected and analysed the data collected through the questionnaires administered regarding alcohol and sleep habits.

### 2.2. Assessment of Alcohol Consumption

Alcohol use was assessed using a short form of the Alcohol Use Disorders Identification Test-Concise (AUDIT-C), a modified version of the 10-question Alcohol Use Disorders Identification Test (AUDIT) developed by the World Health Organization. This test is a brief self-reported alcohol screening test effective for assessing unhealthy alcohol use. This instrument is a 3-item survey with a total score ranging from 0 to 12 points. Each item has five response options rated from 0 points to 4 points. A score of 3 or more points on the AUDIT-C could indicate people who are at-risk drinkers or have alcohol use disorders. A score of 4 for men and 3 for women or more for each is considered predictive of potential alcohol abuse. Commonly, the likelihood of a person having an alcohol use disorder is directly proportional to the highest score on the test.

### 2.3. Assessment of Insomnia Disorder

The assessment of insomnia was conducted through the administration of the Insomnia Severity Index (ISI), a brief validated instrument that can assess the severity of both nocturnal and diurnal components of insomnia. This test is a self-report questionnaire that assesses the nature, severity, and impact of insomnia. The time period to which the test refers is the “last month” and the parameters assessed are severity of sleep onset time, sleep maintenance, morning awakenings, sleep dissatisfaction, interference of sleep difficulties with daytime functioning, visibility of sleep problems by others, and distress caused by sleep difficulties. This instrument consists of a 7-item survey (5 potential responses each) with a total score ranging from 0 (no problem) to 4 (very severe problem). The total score is interpreted as follows: no insomnia (0–7); subthreshold insomnia (8–14); moderate insomnia (15–21); and severe insomnia (22–28).

### 2.4. Statistical Analysis

Statistical analysis was performed using the Graph Pad Prism 8.01 statistical software package (San Diego, CA, USA). The data were tested for normal distribution using the D’Agostino & Pearson omnibus normality test. Because of their normal distribution, statistical analysis was performed with a parametric test as reported. Differences in Alcohol Use Disorders Identification Test-Concise and Insomnia Severity Index scores between day and night workers were analyzed using Student’s two-tailed *t*-test for unpaired data.

For data on the correlation between AUDIT-C and ISI score, we performed a Pearson’s correlation coefficient test. Simple linear regression analyses were generated as predictive models to assign the correlation found. To account for potential confounding factors, the variables gender and age were analysed by employing the General Linear Model—Multivariate Analysis of the Statistical Package for Social Sciences (International Business Machines Corp. IBM, Armonk, NY, USA, version 28). Data were reported as mean ± SD or mean with 95% CI Statistical significance was set at *p* < 0.05.

## 3. Results

### 3.1. Alcohol Consumption

**Figure 2:**
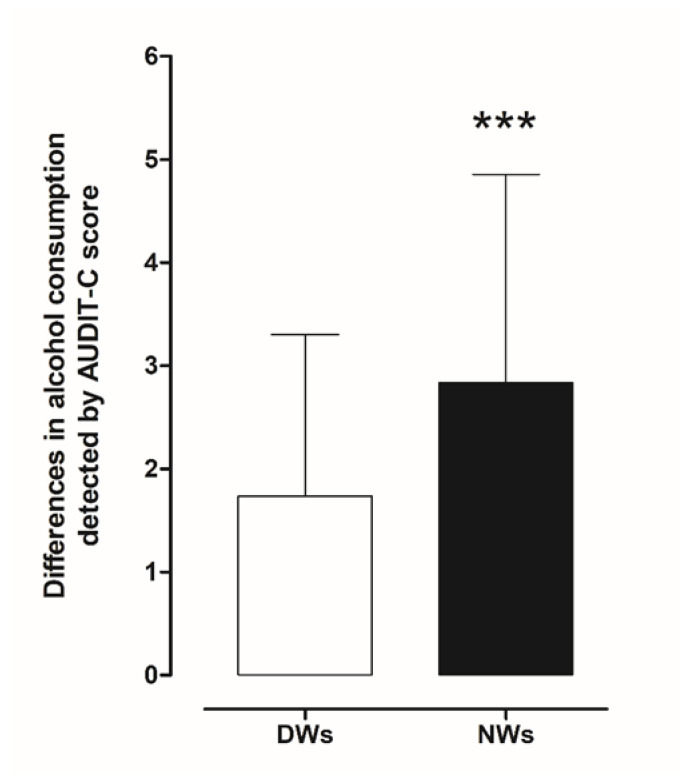
Comparison of differences in AUDIT-C scores between day and night workers. Each value represents the mean ± S.D. of one hundred and six workers. *** *p* < 0.001 compared to daily workers. DWs, daily workers; NWs, night workers.

Assessment of the frequency of alcohol consumption, the average amount of alcohol consumed, and the frequency of consumption of large amounts of alcohol during a short period of time (four to six units) was conducted using AUDIT-C. Initially, we estimated differences in risky drinking (drinking at levels that put a person at risk of medical or social problems) between day and night workers. Statistical analysis using a two-tailed Student’s *t*-test for unpaired measures performed on the mean of the AUDIT-C score showed significant risky drinking behaviour in NWs (t = 6.034, df = 390, *p* < 0.001) compared to DWs (Figure 2).

In more detail, a total of 162 NWs (82.7%; audit score (AS) 3.383, confidence interval (CI) 3.119–3.646) reported alcohol intake compared to 136 (69.4%; AS 2.5, CI 2.284–2.716) of DWs; 129 NWs (65.8%; AS 2.876, CI 2.638–3.144) reported occasional drinking, 18 (9.2%; AS 4.8, CI 3.983–5.462) drank weekly and 16 (8.2%; AS 6.1, CI 5.607–6.518) drank daily compared to DW 121(62.7%; AS 2.2, CI 2.058–2.421), 12 (6.1%; AS 4.4, CI 3.541–5.293), 3 (1.5%; AS 5.3, CI 3.899–6.768).

**Figure 3:**
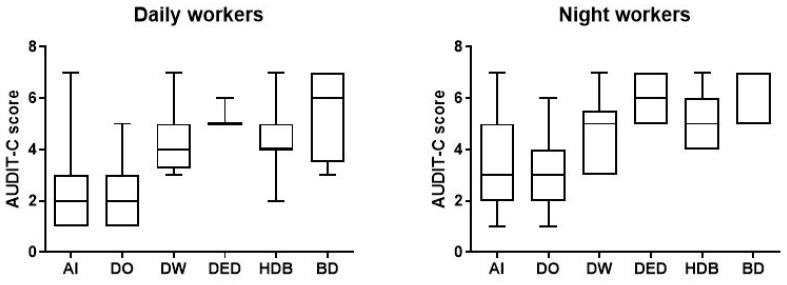
Data on alcohol consumption of the 196 day and night workers enrolled in the study. Above each bar is indicated the number of subjects belonging to the specific group. AI = alcohol intake; DO = drink occasionally; DW = drink weekly; DED = drink every day; HDB = harmful drinking behaviour; BD = binge drinking.

In addition, according to the AUDIT-C criteria, we identified 66 workers (33.7%; AS 5.0, CI 4.789–5.302) with harmful consumption in the NW group and 25 (12.8%; AS 4.40, CI 4.009–4.871) in DW group. We also found 15 NWs (7.7%; AS 5.7, CI 5.244–6.233) and 4 DWs (2%; AS 5.5, CI 2.453–8.547) tended to consume greater amounts of alcohol per occasion (Figure 3) (Table 2).

When AUDIT-C score was controlled for gender and age variable, the analysis indicated no significance impact of gender (*p* = 0.882) and age (*p* = 0.072).

### 3.2. Insomnia Severity Index (ISI)

**Figure 4:**
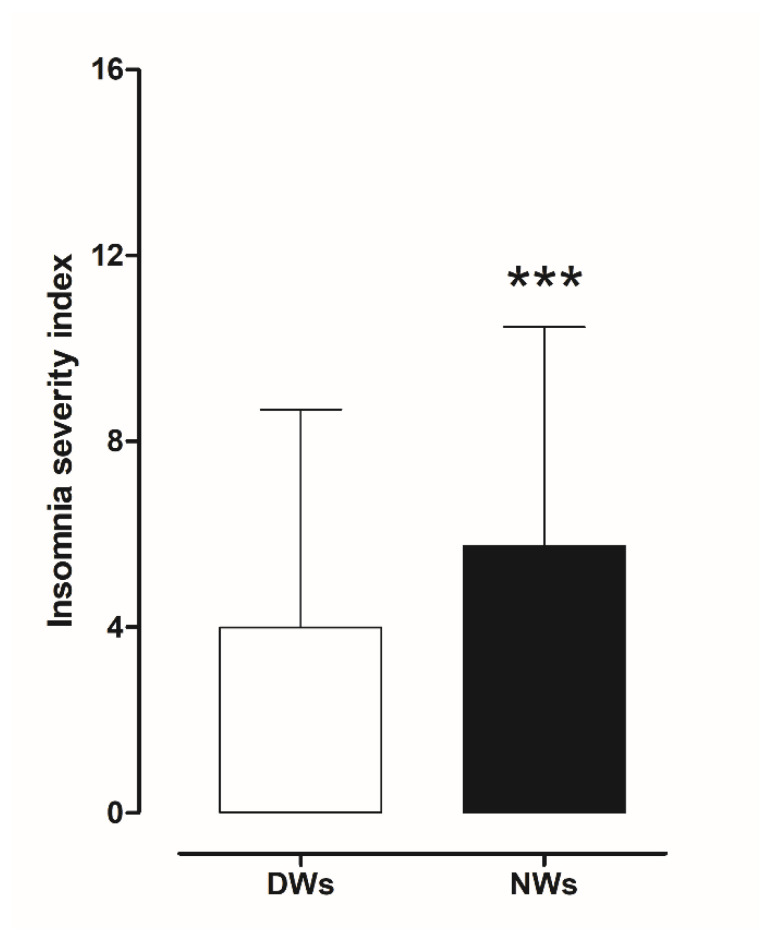
Differences in insomnia severity index score between day and night workers. Each value represents the mean ± S.D. of one hundred and six workers. *** *p* < 0.001 compared to daily workers. DWs, daily workers; NWs, night workers.

The results obtained from the ISI scores were evaluated to investigate the presence of insomnia disorder among day and night workers. Statistical analysis using a two-tailed Student’s *t*-test for unpaired data, performed on the mean of the total ISI score, showed a significant worsening of sleep quality in NWs (t = 3.0706, df = 390, *p* < 0.002) compared to DWs (Figure 4).

To understand the degree of insomnia, we also divided the sample populations in relation to total score categories and analysed the difference between day and night workers.

**Figure 5:**
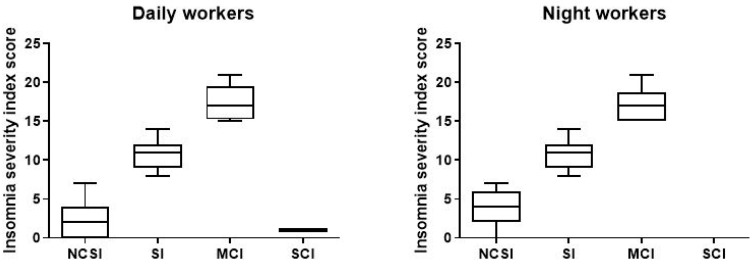
Data on sleep problems of the 196 day and night workers enrolled in the study. Above each bar is indicated the number of subjects belonging to the specific group. NCSI = no clinical significant insomnia; problems; SI = subthreshold insomnia; MCI = clinal insomnina; SCI = severe clinical insomnia.

As shown in in the picture (Figure 5), among the NWs participants in the study, a total of 151 (77%; ISI 3.63, CI 3.266–3.992) reported no sleep problems, compared to 167 DWs (85.2%; ISI 2.63, CI 2.027–2.619); 29 (14.8%; ISI 10.59, CI 9.90–11.27) had subthreshold insomnia, and 16 (8.2%; ISI 17.06, CI 15.92–18.20) resulted in moderate clinical insomnia compared to DWs (7.7%; ISI 10.47, CI 9.466–11.47), 12 (6.1%; ISI 17.33, CI 15.82–18.75). We find only one (0.51%) DWs with severe clinical insomnia (Table 3).

When ISI score was controlled for gender variable, the analysis indicated no significance impact of gender (*p* = 0.661) and age (0.083).

### 3.3. Alcohol Consumption and Insomnia Disorder

**Figure 6:**
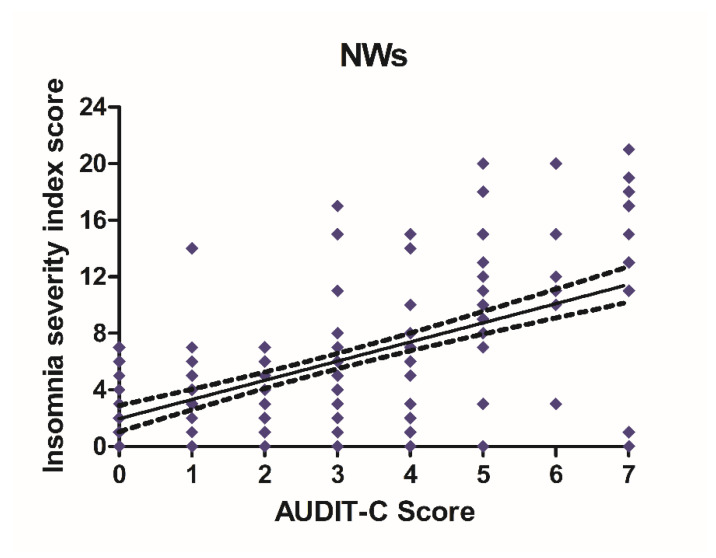
Correlation between AUDIT-C and ISI score in night workers. Each value represents the mean ± S.D. of one hundred and ninety-six workers. DWs, daily workers; NWs, night workers.

**Figure 7:**
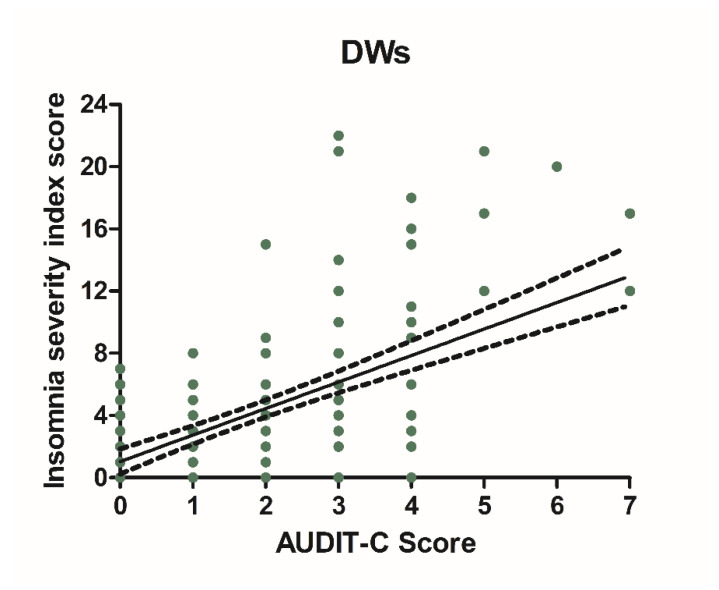
Correlation between AUDIT-C and ISI score in night workers. Each value represents the mean ± S.D. one hundred and ninety-six workers. DWs, daily workers; NWs, night workers.

Alcohol consumption and insomnia severity problems were assessed to evaluate a presumed influence of alcohol use with the onset of sleep disorders. For this purpose, Pearson’s correlation coefficients between AUDIT-C score and ISI score were performed. A significant positive correlation was found between sleep problems and alcohol use in NWs (r = 0.5781, CI 0.4766–0.6645, *p* < 0.0001) (Figure 6) and DWs (r = 0.5702, CI 0.4674–0.6578, *p* < 0.0001) (Figure 7).

## 4. Discussion

The present research sought to examine differences in alcohol consumption and insomnia disorder among work groups with different work schedules. In addition, we investigated the links between alcohol consumption and insomnia. Using a questionnaire method to estimate alcohol consumption and risk behaviours [50], we found that, among daily workers, 22.4% had alcohol-related problems and that working primarily at night negatively influenced the occurrence of alcohol-related problems, resulting in 58.8% of subjects having alcohol-related problems. Specifically, we found that, among people who work primarily at night, there were 33.7% of workers who engaged in harmful drinking behaviour, and 7.7% reported binge-drinking in the past year.

The results of this study are in agreement with some research showing that night workers are more likely to consume alcohol than day workers, often in a pattern of binge drinking [35,36]. These differences are likely due to the working conditions night workers are subjected to, which make them particularly vulnerable to stress. It is well established that particularly dangerous and demanding work, especially when performed at night, is a major stressor on the body and can affect the worker’s overall health and well-being [26,51,52]. It is also important to remember that, at a biological level, the interruption and reversal of the sleep/wake cycle, together with the modified activity/rest pattern, can generate particularly stressful living conditions, making the night worker more vulnerable to stress-related disorders [53,54,55,56,57].

Stress, generally defined as any stimulus that disturbs the body’s homeostasis, is an important predisposing factor to uncontrolled alcohol consumption [58,59]. On the other hand, the intake of alcohol is often related to its ability to alleviate a particular state of functional discomfort that leads the subject to constantly take the substance up to dependence [60,61]. The mechanisms that correlate stress with alcohol intake can be traced to their different modulation of the hypothalamic-pituitary-adrenal (HIP) axis [62,63]. Under stressful conditions, cortical and subcortical centers activate the hypothalamic paraventricular nucleus, promoting the secretion of corticotropin-releasing hormone (CRH) which, in turn, stimulates the release of adrenocorticotropic hormone (ACTH) by the adenohypophysis, causing the secretion of cortisol from the adrenal glands [64,65]. CRH is also capable of promoting increased levels of catecholamines (noradrenaline and dopamine) in certain extra-hypothalamic regions such as the amygdala and prefrontal cortex. This increase, along with that of glucocorticoids, is necessary for our brain both to understand the rewarding or aversive value of a substance and to learn the potential of its reinforcement, such as the learning associated with alcohol intake as a stress management mechanism [66,67,68,69].

Alcohol intake is able to modulate the same circuits that underlie the adaptive response to stress [70,71]. It has been shown that, after acute exposure to stress or moderate doses of alcohol, dopaminergic and hypothalamic circuits recover their normal basal tone allowing a normal response to novel stimuli [72]. In contrast, under conditions of intense stress or when high doses of alcohol are consumed, systems undergo a set of functional adaptations of reward and neuroendocrine regulatory circuits [73,74] that may mark the pathophysiological change underlying the transition from controlled seeking to compulsive drinking [75,76]. Furthermore, binge drinking is able to generate tolerance to both stress and alcohol intake [71,77,78].

The above can partly explain the results obtained in our research. Night workers, being subjected to particularly stressful working conditions [79,80], are more likely to consume high amounts of alcohol, probably implementing a coping strategy with respect to the stress they are subjected to on a daily basis during working hours. Moreover, the higher number of binge-drinkers is probably due to a greater sensitivity of some individuals to stress, which makes them more anxious and emotionally unstable and promotes the consumption of large amounts of alcohol in order to extinguish the unpleasant feeling of unbearable stress.

The amount of alcohol consumed by the workers in the study was well correlated with the analysis of of insomnia disorder. Specifically, the results obtained from the insomnia severity index questionnaire showed that there was a higher percentage of night workers (23%) with insomnia than day workers (14.31%). Several studies have shown that there is a bidirectional relationship between alcohol consumption and sleep problems. Specifically, insomnia predispose the subject to the abuse of various substances and, conversely, both acute and chronic consumption of these substances increases sleep disorders [81,82,83,84]. Among substances of abuse, alcohol seems to play a crucial role in the genesis of these disorders [85,86].

A number of studies aimed at understanding the mechanisms underlying workers’ health risks show that the behavioural strategies used to cope with sleep disturbances have a negative impact on their psychophysical integrity. Since workers have to sleep at different times than usual, they use different strategies to induce and improve sleep quality, including alcohol consumption [87,88,89,90].

This molecule has sedative properties and reduces the time needed to fall asleep [91]. However, the prolonged use of alcohol, especially in high concentrations, alters the quality of sleep, both prolonging the time needed to fall asleep and fragmenting the duration of sleep [92]. Finally, it is important to remember that the negative effects of alcohol, especially above recommended limits, are linked to several systemic diseases. Therefore, although perceived as a useful tool for those with sleep disorders, it can be seen as a tool that can promote the disorder itself, further increasing the risk to the health and safety of those who sleep.

The influence of alcohol on sleep architecture is partly attributable to its ability to modulate the activity of several chemicals responsible for nerve transmission. Alcohol acts in a dose-dependent manner by interfering with the action of gamma-aminobutyric acid (GABA) and glutamate, both of which play important roles in the regulation of sleep-wake rhythms [93,94]. Particularly, at low doses, alcohol facilitates the binding of GABA to its receptor site and exerts an antagonistic action on glutamate receptors, promoting an increase in the inhibitory signal and a reduction in the excitatory signal [95] in several brain areas involved in sleep regulation, such as the reticular portion of the brainstem, the thalamus, the hypothalamus, and the basal forebrain [96,97,98]. This alcohol-mediated action may partly explain the motivation of night workers to consume alcohol as a strategy to aid the falling asleep process. This pattern of consumption could also favour the establishment of risky behavioural patterns, which would lead the night worker to consume alcohol in such a way (binge drinking) and with such a frequency as to facilitate the onset of problems related to the use of the substance, as also demonstrated by the results of our research.

The percentage of subjects with alcohol-related problems among night workers compared with day workers may partially explain the high incidence of insomnia disorder found in this group. On the other hand, it has been reported that alcohol, taken in high concentrations and for fairly long periods of time, is able to modify sleep architecture and promote the onset of sleep disorders [86,99,100,101]. It has also been shown that the rate of sleep disturbance is significantly higher in individuals diagnosed with alcohol dependence than in the general population [102,103,104].

## 5. Conclusions

Although this study does not contain a very large sample, it provides further awareness of the importance of the negative impact of alcohol consumption on sleep quality in night workers.

A full understanding of the correlation between alcohol use and insomnia may provide a valuable and effective tool for assessing whether work performance is impaired.

Preventive action through accurate worker history, including the administration of questionnaires to detect the presence of alcohol or sleep disorders, would help both the health of the worker and the increased safety of the worker and those working with them.

Further research aimed at understanding the complexity of the relationship between night work, alcohol use and sleep disorders is timely and important. Alcohol use and the genesis of sleep disturbances can exert a strong negative pressure on already vulnerable physiological systems, leading to the onset of various illnesses and, although less important, decreased work performance.

## Figures and Tables

**Table 1:** Demographic data and other baseline characteristics of patients included in the study. na: number; %: percentage of patients; mean: mean value; SD: standard deviation; Min, Med and Max ranges, respectively.

Male		Percentage	Media	S.D	Min	Med	Max
na	222	56.6%					
Age			45.16	11.77	24	45	67
Height (cm)			1.74	0.079	1.6	1.75	1.91
Weight (kg)			76.9	9.68	59	79	100
BMI			25.25	3.02	18.11	25.29	31.9
**Female**							
na	170	44.4%					
Age			44.94	11.29	27	45	63
Height (cm)			1.68	0.0635	1.58	1.68	1.83
Weight (kg)			67.6	7.65	50	69	82
BMI			23.67	2.39	50	69	29.73

**Table 2:** Data on alcohol consumption of the 196 day and night workers enrolled in the study. All data were calculated from the results obtained from AUDIT-C. n° = number; % = percentage.

	Daily Workers		Night Workers	
	n°	%	n°	%
	196	100	196	100
Workers reporting alcohol intake	136	69.4	162	82.7
Workers who drink occasionally	121	62.7	129	65.8
Workers who drink weekly	12	6.1	18	9.2
Workers who drink every day	3	1.5	16	8.2
Workers with harmful drinking behaviour	25	12.8	66	33.7
Workers who engage in binge drinking	4	2	15	7.7

**Table 3:** Data on sleep problems of the 196 day and night workers enrolled in the study. All data were calculated from the results obtained from the Insomnia Severity Index score. n° = number; % = percentage.

	Daily Workers		Night Workers	
	n°	%	n°	%
	196	100	196	100
No clinically significant insomnia	167	85.2	151	77
Subthreshold insomnia	15	7.7	29	14.8
Moderate clinical insomnia	12	6.1	16	8.2
Severe clinical insomnia	1	0.51	-	-

## Data Availability

The data is not publicly available due to privacy restrictions.
